# Financial Literacy Among Medical Trainees and Faculty: A Pilot Study

**DOI:** 10.7759/cureus.44829

**Published:** 2023-09-07

**Authors:** Adeel S Zubair, Dinesh K Sivakolundu, Marcus DeVito, Jeffrey J Dewey

**Affiliations:** 1 Neurology, Yale School of Medicine, New Haven, USA; 2 Finance, National Financial Network, New Haven, USA; 3 Neurology, Yale New Haven Hospital, New Haven, USA

**Keywords:** finances, attending, fellow, resident, financial literacy

## Abstract

Introduction: Medical education systems are trained to produce efficient, thorough clinicians. These programs provide limited training on personal finances. The current socioeconomic climate for medical trainees includes increasing education debt and stagnating reimbursement. We conducted a survey-based cross-sectional pilot study at an academic institution targeted at residents, fellows, and attendings of all medical specialties. Our aim was to understand baseline levels of financial literacy at different training and career stages, which can inform targeted interventions to improve this crucial aspect of physician well-being.

Methods: A survey was devised with the assistance of a certified financial planner. This survey was distributed at an academic institution targeting residents, fellows, and attendings. The survey was anonymous, and no identifying data were collected. Two reminders were sent to subjects to complete the survey.

Results: A total of 50 physicians completed the survey in 2021. There were eight responses from interns, 14 responses from residents (post-graduate year 2 or later), 14 responses from fellows, and nine responses from attendings. The majority of our respondents reported not having any particular financial education, and over 70% of respondents reported that their graduate education had not provided them with the tools needed for personal financial success.

Conclusion: Financial education and financial literacy are important topics that need to be further incorporated into the medical education pathway. Physicians are not well equipped in this realm, and further training is necessary. This study provides pilot data that highlight important aspects of physician knowledge and practices in regard to finances.

## Introduction

Throughout the medical education system, a strong emphasis is placed on building core foundations for basic science and clinical knowledge [1-3]. In conjunction, students are taught about healthcare policy, care delivery models, and barriers to healthcare [4-7]. However, little to no instruction is given on the necessary skills for work-life integration such as financial planning. Medical students, resident physicians, and attending physicians have historically had limited financial literacy and have been disadvantaged in financial education compared to their peers in other careers [8-10]. These medical professionals spend a lot of time training before they reach the stage where they make their full income. Additionally, during this time, they incur significant loans and debt, which makes for a long and arduous process to become financially independent.

More recently, there has been an additional focus on well-being and preventing medical professional burnout [11]. Personal finances and financial education are important cornerstones of physician satisfaction and wellness. The current socioeconomic climate for medical trainees includes increasing education debt and stagnating reimbursement. As a result, financial education is a crucial part of the medical school and residency curriculum, which is currently lacking. Studies have shown that debt negatively impacts medical student career choices, career satisfaction, and overall quality of life [12-14]. Additionally, debt has been linked with burnout, irrespective of specialty or level of training [15-18].

We conducted a survey-based pilot study at an academic institution targeted at residents, fellows, and attendings of all medical specialties. Our aim was to understand baseline levels of financial literacy at different training and career stages, which can inform targeted interventions to improve this crucial aspect of physician well-being.

## Materials and methods

A survey was devised with the assistance of a certified financial planner (Supplementary Material). This survey was used to collect general demographic information coupled with financial demographics and financial literacy information. Additionally, the final part of the survey was aimed at understanding respondent views toward their financial education and the impact their finances have on their lives and careers.

This study was approved by the Yale Institutional Review Board (2000029853). It was distributed to the resident physicians, fellows, and attending physicians through the graduate medical education department at Yale via the Qualtrics online survey portal and email. The sample group of the study was intended to be broad to create representative data. The survey was anonymous, and no identifying data were collected. Two reminders were sent to subjects to complete the survey.

## Results

A total of 50 physicians completed the survey in 2021. All residents and fellows received the study, as did attendings who worked with graduate medical education. There were eight responses from interns, 14 responses from residents (post-graduate year 2 or later), 14 responses from fellows, and nine responses from attendings. The respondent pool was roughly 60% male (30 responses). Of the respondents, 31 (62%) were White, four (8%) were Black, and 11 (22%) were Asian. Forty-one (82%) subjects majored in sciences. Only one majored in business as an undergraduate.

**Figure 1:**
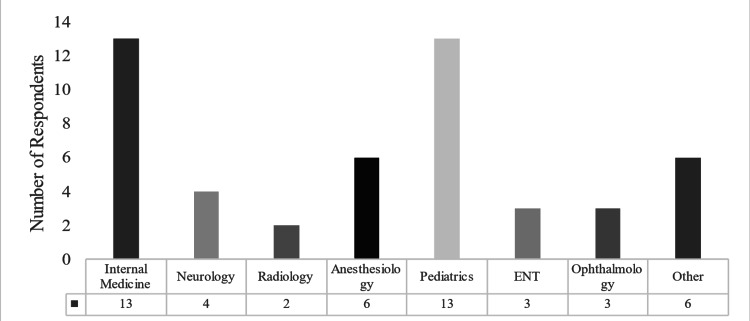
Breakdown of participants by medical specialty.

Of the medical specialties, Internal Medicine and Pediatrics were the specialties with the highest responder count of 13 in each, followed by Anesthesiology with six respondents and Neurology with four (Figure 1). The majority of respondents lived with a significant other (46.3%), while 18.5% lived by themselves and 31.5% lived with family. Thirty-two percent of the subject pool had children.

When rating their financial knowledge, 44% of subjects responded that they felt that they were at the beginner level, with only 26% reporting that they were experienced or at the expert level. Sixty percent of the cohort reported that they had student debt. Of those who stated they had student debt, 21.4% had greater than $275,000, while 25% had between $200,000 and $275,000 and 21% had between $125,000 and $200,000. In addition, 23.1% reported that they had a mortgage, 24.6% reported a car loan, and 15.4% reported credit card debt. 

We sought to assess how one’s financial knowledge influenced their financial management, decisions, stability, and wealth. To test this hypothesis, we compared variables that reflect financial management (i.e., use of an accountant or financial advisor, etc.), decisions (i.e., formalized monthly budget, etc.), stability (i.e., emergency fund, etc.), and wealth (i.e., net worth) between individuals who rated themselves as beginners in financial knowledge (i.e., beginners) and those who rated themselves as intermediate or experienced in financial knowledge (i.e., advanced). We found that a higher proportion of individuals with advanced knowledge use an accountant (x^2^(1)=5.1165, p=0.024), contribute regularly to retirement accounts (x^2^(1)=6.9274, p=0.008), have active disability insurance (x^2^(1)=0.72323, p=0.007), have an emergency fund (x^2^(1)=4.1574, p=0.041), and purchase stocks and follow market trends (x^2^(1) =11.3673, p=0.001) compared to those individuals with beginner knowledge. 

Next, we investigated the effect of income status on financial management, decisions, stability, and wealth. We compared these variables between those with a family income less than $100,000 (i.e., low income) and those with a family income greater than $100,000 (i.e., high income). We found that high-income individuals are more likely to have an accountant (x^2^(1) =10.5237, p=0.001), less likely to be a primary decision-maker (x^2^(1) =4.6557, p=0.031), and less likely to prepare their own taxes (x^2^(1) =12.5335, p<0.001) compared to those with low income.

We also assessed the effect of credit scores on financial management, decisions, stability, and wealth. We found that those with high credit scores were more likely to be the primary decision-maker (x^2^(2)=7.9432, p=0.019) and prepare their own taxes (x^2^(2)=7.6077, p=0.022) and were less likely to purchase stock and follow market (x^2^(2)=12.777, p=0.002), use an accountant (x^2^(2)=14.8950, p=0.001), or take courses on finance or business (x^2^(2)=7.8458, p=0.020).

**Figure 2:**
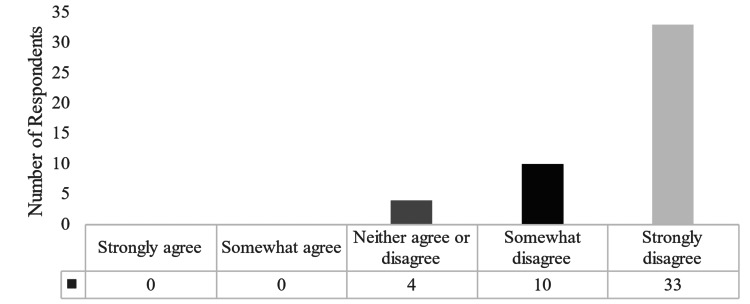
Participants reported confidence in their personal financial preparedness through their medical education.

The majority (85.1%) of subjects reported that they have not taken any courses on finance or business. When asked about their graduate education providing information regarding personal finances, 70.2% strongly disagreed that their graduate education had provided them with the tools needed for managing personal finances (Figure 2). The majority (68.3%) of subjects reported that their career decisions are somewhat influenced by financial considerations, whereas 17% reported that they are primarily influenced by financial considerations.

## Discussion

Most graduate medical education programs have a limited focus on financial education and financial literacy, and as a result, students and physicians-in-training must often learn either through real-world experience or via self-guided methods. The importance of financial education cannot be minimized; many students graduating from medical education programs have some level of student debt, and poor financial literacy has been linked with higher rates of burnout [16-18]. As a result, identifying these deficiencies and focusing on improvement is important for improving provider wellness as well as the longevity of their careers [15-18]. Adequate financial literacy can help minimize the risk of financial complications for physicians.

This study provides pilot data from a single cohort of trainees at an academic hospital. Conforming with most reported data, the majority of our population included those who had majored in the sciences as undergraduates [19,20]. Only one member of our subject pool reported that they had majored in a financial discipline. Forty-four percent of our pool reported a beginning level of financial knowledge. The majority of our subjects reported they had not taken any particular courses in finance for business and did not have a financial advisor (72.3%) or an accountant (74.5%). The vast majority of our subjects are the primary decision-makers in regard to their finances (89.4%). Lastly, over 70% of respondents strongly disagreed that their graduate education had provided them with the tools needed regarding their personal finances.

Our study cohort had a variety of different levels of debt, with over 60% of our subjects reporting greater than $100,000 in debt. Other sources of debt were mortgages, car loans, and credit card debt. These loan burdens were reported to play a role in career decision-making. 

Additional notable findings from this study include the fact that the majority of physicians do not use a financial advisor or an accountant. Our study showed that a higher proportion of individuals with advanced knowledge were likely to use an accountant, contribute regularly to retirement accounts, have active disability insurance, have an emergency fund, and purchase stocks and follow the market compared to those with beginner knowledge. We additionally identified that high-income individuals are more likely to have an accountant, less likely to be a primary decision maker, and less likely to prepare their own taxes compared to those with low income. These decisions can have significant downstream effects when it comes to financial planning and tax strategy and can lead to a longer time until financial independence. 

These findings highlight that while financial literacy is important in medical education and physician lives, little education is provided in this area during medical training. Our cohort identified that they do not feel proficient in financial management and have not received any particular training in regard to their finances. Given the significant amount of debt that physicians carry, coupled with the fact that their training in this area is limited, it is important to empower physicians to make financially sound decisions.

Limitations of the study included the small sample size, the focus on one academic center, and responder bias. Those physicians who were on either extreme of financial literacy may have been missed in this cohort due to self-selection. Additionally, the vast majority of respondents to the study were in medical specialties, with very few surgical specialties represented. With those limitations in mind, the study corroborates other studies highlighting the inadequacy of financial education in the medical training pathway and the importance this plays in the lives of physicians. Further, larger, multi-institutional studies will help further gather data regarding this important topic and can help lead to curriculum change.

## Conclusions

Financial education and financial literacy are important topics that need to be further incorporated into the medical education pathway. Physicians are not well equipped in this realm, and further training is necessary. This study provides pilot data that highlight important aspects of physician knowledge and practices in regard to finances. Further studies are necessary to expand on these topics identified and to help guide change in the medical education system.

## Appendices

Demographics

1.   Type of student/provider (law, business, medical student, resident, fellow, attending)

2.   Age

3.   Gender

4.   Race

5.   Undergraduate major (primary): science, economics/business/finance, humanities, other

6.   Year in school/training (college freshman=year 1, 1-70)

7.   For resident/fellow/attending, specialty of practice

8.   Current state of residence

9.   I live with (myself/roommates/significant other/family)

10. I have children in my household (yes/no)

a.  If yes, number

Financial demographics

1.   I would rate my financial knowledge as: beginner to expert

2.   I have student debt (Y/N)

a.   If yes, amount of student debt

  i.   $1-$25,000

  ii.  $25,000-$75,000

  iii.  $75,000-$125,000

  iv.  $125,000-$200,000

  v.   $200,000-$275,000

  vi.  $275,000+

3.   Other sources of debt

a.   Mortgage

b.   Car loan

c.   Credit card debt

d.   Home equity loan

4.   Annual income?

a.   $45,000-$70,000

b.   $70,000-95,000

c.   $95,000-140,000

d.   $140,000-200,000

e.   $200,000-260,000

f.    $260,000+

5.   What is your credit score (unknown, excellent 800-850, very good 740-799, good 670-739, fair 580-669, very poor 300-579)?

6.   I have a financial advisor.

7.   I have an accountant.

8.   I am the primary decision-maker in your finances.

9.   I prepare my own taxes.

a.  If no, someone else in a family household, or an accountant?

Financial literacy questions

1.   I contribute regularly to my 401k/403b retirement account.

2.   I take advantage of pre- and post-tax retirement accounts.

3.   I have an emergency fund that would cover three to six months of essential expenses.

4.   I have an active life insurance policy.

5.   I have an active disability insurance policy.

6.   I have a formalized monthly budget.

7.   Over the last 12 months, I have studied my budget (x months).

8.   I calculate my net worth regularly (If yes, how frequently (weekly/monthly/yearly)).

9.   I have an established plan to reduce my debt (if yes, see below).

a.   I am trying to pay off my debt sooner than scheduled.

10.  I regularly purchase stocks and follow the stock market.

11.  I have taken courses on finances/business.

Other questions

1.   I feel my graduate education provided me with adequate information and training regarding personal finance.

2.  My career decisions are (not at all/somewhat/primarily) influenced by financial considerations.

3.   I am concerned about my current debt (not at all/somewhat/significantly).

4.   I learned about finances through (courses, family, friends, on the job, self-taught via Internet, books, etc., other, none of the above).
